# ‘Complex’ but coping: experience of symptoms of tuberculosis and health care seeking behaviours - a qualitative interview study of urban risk groups, London, UK

**DOI:** 10.1186/1471-2458-14-618

**Published:** 2014-06-18

**Authors:** Gillian M Craig, Louise M Joly, Alimuddin Zumla

**Affiliations:** 1 School of Health Sciences, City University London, Northampton Square, London, EC1V 0HB, UK; 2 Department of Infection, Division of Infection and Immunity, Centre for Clinical Microbiology, University College London, and NIHR Biomedical Research Centre, University College London Hospitals, London, UK

**Keywords:** Tuberculosis, Social determinants of health, Health care seeking behaviour, Tuberculosis, Homeless persons, Drug users, United Kingdom

## Abstract

**Background:**

Tuberculosis awareness, grounded in social cognition models of health care seeking behaviour, relies on the ability of individuals to recognise symptoms, assess their risk and access health care (passive case finding). There is scant published research into the health actions of ‘hard-to-reach’ groups with tuberculosis, who represent approximately 17% of the London TB caseload. This study aimed to analyse patients’ knowledge of tuberculosis, their experiences of symptoms and their health care seeking behaviours.

**Methods:**

Qualitative interviews were conducted with 17 participants, predominantly homeless and attending a major tuberculosis centre in London, UK. Most had complex medical and social needs including drug and alcohol use or immigration problems affecting entitlement to social welfare. Analytical frameworks aimed to reflect the role of broader social structures in shaping individual health actions.

**Results:**

Although participants demonstrated some knowledge of tuberculosis their awareness of personal risk was low. Symptoms commonly associated with tuberculosis were either not recognised or were attributed to other causes for which participants would not ordinarily seek health care. Many accessed health care by chance and, for some, for health concerns other than tuberculosis.

**Conclusions:**

Health education, based on increasing awareness of symptoms, may play a limited role in tuberculosis care for populations with complex health and social needs. The findings support the intensification of outreach initiatives to identify groups at risk of tuberculosis and the development of structured care pathways which support people into prompt diagnosis and treatment.

## Background

Tuberculosis (TB) was once considered to be a disease of the past in the United Kingdom in the early 1980s and TB services were reduced to a minimum. It was only in the late 1990s that there was a growing awareness about the rising incidence of TB in urban environments in major cities in the UK and Europe [1-3]. Cases of TB are over-represented in socially and economically marginalised groups in high income countries. Groups that are affected by TB in the UK include migrants from high TB endemic countries, homeless people, prisoners, people living with HIV (PLHIV) and people who use drugs (PWUD) and alcohol [3,4]. These groups are at greater risk of TB than the general population. They also comprise: 38% of non-treatment adherent cases, 44% of cases lost to follow up, 30% of cases deemed highly infectious and represent approximately 17% of the London TB caseload [5]. Tuberculosis services within the NHS in the UK may spend a disproportionate amount of time and effort supporting individuals through the six months of TB treatment because of co-morbidities and several social risk factors affecting patient adherence to taking TB medication.

The cornerstone of TB care relies on early detection and diagnosis of disease in patients (case finding), screening of their contacts (contact tracing) and treatment completion (case holding). An estimated three million people with active TB remain undiagnosed each year globally; continue to spread the disease, with major implications for their own health, and the health of communities they live in [6]. Public health services rely largely on passive case finding which assumes that people have an awareness of TB, are able to recognise their symptoms and seek treatment.

Much of the health care seeking behaviour literature has been dominated by social cognition models such as the Health Belief Model. This was initially developed to understand the reasons for the failure of a free, preventative TB screening programme in the US in the 1950’s [7]. This model broadly suggests that people will seek health care based on their perceived risk of contracting tuberculosis, the severity of the disease and its consequences. The likelihood of the patient seeking help depends on his or her perception of the benefits of pursuing a course of action and any perceived barriers. Additional ‘cues to action’ are predicated on knowledge of symptoms and ability to act on those symptoms (self-efficacy). Acknowledging that this model may be over simplistic, a further distinction has been posited between deliberative choices and actions, which are consciously planned, and non-deliberative which are habitual and less amenable to rational thought or changes in behaviour [8]. The UK House of Lords Science and Technology Select Committee, tasked to analyse the evidence underpinning polices aimed at changing behaviour for example, noted that much research has emphasised the deliberative, rational actor approach at the expense of non-deliberative health actions [8]. Further criticism levied at social cognition models concerns their conceptual basis, which is not strong, [9] and lack of attention to the role of context in shaping decisions about health care [10]. For example it is widely recognised that those who are in most need of healthcare are least able to access services, a phenomenon termed the ‘inverse care law’ [11].

Adopting a structural approach to inequality, the work of Paul Farmer has emphasised the economic, political and institutional contexts which underscore behaviour and the risk of infectious diseases. Farmer argues that much research and clinical care overstates individual agency at the expense of social factors which can affect an individual’s ability to seek health care and adhere to a course of treatment [12,13].

Farmer’s emphasis on structures also resonates with a social determinants model of health which has recently entered policy discourse as a framework for understanding health inequalities and tuberculosis [14]. The emphasis on a life course approach further highlights how health outcomes are shaped by the social structures and economic environments in which people: “are born, grow, live, work and age” [15,16]. It is within these frameworks and debates about how individual actions (agency) are structured in everyday contexts that we attempt to situate the experiences of our participants and our analysis.

Research in the UK has focussed on the socio-cultural understandings of TB in migrant groups, including the Somali [17] and African [18] communities. However, there is scant published qualitative research which analyses the experiences of marginalised groups. Insight into vulnerable groups’ awareness of TB and health actions may assist services to develop initiatives that promote early contact and develop structured and supportive pathways into care. We thus examined the assumptions underpinning passive case finding.

Our interview study formed part of a wider service development project conducted in London, UK, which aimed to develop a social outreach model of care for marginalised groups with TB and generate an evidence base for the need of a TB caseworker in supporting clients with complex needs and is reported elsewhere [19,20].

## Methods

The analysis was informed by a critical health psychology perspective which understands illness behaviour within social, political and cultural contexts which not only influence health and illness, but systems of health care [21-23]. We drew on Wilkinson and Marmot’s ‘key facts’ model to situate participants’ experiences within structures which both place individuals at risk and shape health actions, including: stressful life circumstances, influence of early life, social exclusion, unemployment, addiction, food, opportunities for healthy lifestyle and access to services [16].

### Study participants

Participants in the interview study were recruited from a major TB centre characterised by a culturally diverse catchment area including migrant, homeless and drug using populations. Interviewees were selected for inclusion based on a risk assessment, completed by nurses, and was designed specifically for the service development initiative [20]. The risk assessment identified those health and social risk factors likely to complicate adherence to treatment (such as homelessness and drug use) and eligibility for referral to a TB caseworker for enhanced case management and support. Sampling was broadly purposive and reflected a range of ‘critical case’ experiences typical of those presenting with complex needs within the clinic and the caseworker’s caseload [24,25]. Participants were informed that the study was part of a new initiative evaluating the role of a TB caseworker in developing collaborative care pathways. Participants were referred to the researcher by nurses or the case worker based on the patient’s risk assessment.

Interviews lasted between 30 and 60 minutes and covered broad based themes about the experience of health and illness, knowledge of TB, symptoms and access to health care including any barriers. The majority of interviews took place in the hospital outpatients’ clinic, three took place on a hospital ward, one interview took place in a homeless hostel and one in a prison with the permission of the managers in charge. Clinic interpreters were used in two cases. Interviews generally coincided with patients’ clinic appointments and they were offered a food voucher to the value of £5. Due to the difficulty of researching this group within a clinical environment (e.g. lack of private spaces and frequent interruptions), participants were interviewed on more than one occasion over the course of their treatment. For example, interviews were rescheduled at the request of the patients if they needed to leave the hospital premises (to smoke or drink for example), or when they were called to see the doctor or nurse. All interviews were audio recorded and transcribed verbatim, except for the two interviews involving interpreters and the one in the prison. Here the researcher took notes.

### Analysis

Our approach was a theoretical thematic analysis [26]. Coding involved three stages including deductive (top down) and inductive (bottom up) coding and linking codes to our theoretical frameworks. The first level of coding began with readings of the transcript to identify segments of relevant text relating to knowledge of TB, recognition of symptoms of TB, and examples of how participants accessed care and contextual information about individual life experiences. These were then compared across transcripts (stage 2). Our top down analysis involved linking our codes to our social determinants. Analytic memos were used to aid analysis. Coding was compared between researchers (GC, LJ), one with a social science background (GC) and one with a background in nursing and homelessness (LJ). Data analysis was managed using a computer software programme designed specifically for the coding and retrieval of qualitative data (QSR NUDIST*Vivo 10).

### Ethical considerations and consent

This research was carried out within the guidelines of University College London Hospital’s ethics committee which approved the study. Written consent was obtained where possible by the researcher (GC). Where participants were unwilling, or unable, to provide written consent (for example due to arthritis), verbal consent recorded as part of the interview was accepted (as agreed with the ethics committee). Participants were advised that the interview did not form part of their clinical care and would in no way affect their entitlement to treatment. If the researcher saw that the participant was becoming unwell or agitated, the interview was terminated and rescheduled. Where participants became distressed or disclosed distressing experiences, the researcher offered to terminate the interview and refer them to a support organisation or to the TB case worker.

## Results and discussion

### Participant characteristics

We interviewed 17 participants: sixteen individuals with a confirmed diagnosis of TB and one with suspected tuberculosis. The majority of interviewees were male (71%; 12/17). Just over half were born in the UK (59%; 9/17) and of these six described their ethnicity as White British. The remainder were: two of Irish origin; one Black British; two Black African; and, one woman who described her ethnicity as Jewish. Of those born outside the UK, two were Ethiopian and three Somalian. The mean age of respondents was 44 years (range 18–67; n = 16) at the start of their initial treatment.

### Income, housing and employment

Five participants had complex immigration cases affecting their entitlement to housing and welfare. For example, three participants (migrants) were engaged in formal employment prior to their diagnosis (catering industry, sales assistant and a health care worker) but became unemployed due to illness and were therefore left without an income and with no recourse to public funds. One homeless man engaged in casual labour on a market another relied solely on begging for income. The remainder were in receipt of some form of benefit, voucher scheme (used in exchange for food in designated shops) or subsistence support of £30 per week. Two women had engaged in sex work. Table 1 illustrates the housing status of participants at the time of interview. The majority would be described as homeless according to statutory legislation.

**Table 1:** Social characteristics of interviewees

**Int ID**	**Usual pattern of housing+**	**Place of birth**	**Criminal justice**	**Drug use**	**OST**	**Alcohol**	**HIV+**	**Other self reported health conditions**	**Routes into care**
ID01	Hostel	UK	✓	PWID	✓	✓		Epilepsy	Unclear due to several attempts to treat over a period of time. Not hospitalised.
ID02	NFA	UK	✓			✓		Epilepsy	GP called ambulance (A & E)
ID03	Shared house (temporary)	Ethiopia							Awaiting referral via GP but self referred to A & E and hospitalised
ID04	BedsitHostel	Nigeria	✓	PWUD		✓		Diabetes	Blacked out, taken to hospital by ambulance, admitted to hospital through A & E
ID05	NFA	UK		PWID	✓				MXU & hospitalised
ID06	NFA	UK	✓	PWID	✓		✓		Collapsed outside hospital, self referred to A & E & hospitalised initially in a psychiatric unit because of drug use
ID07	B & B	Somalia						Hypertension	GP referred patient to TB clinic
	Staying with relatives in their house								
								Diabetes	
								Ulcer	
ID08	Home ownership but now unable to pay mortgage due to illness and no recourse to public funds due to immigration status	Nigeria					✓	Drug induced diabetes	Friend called ambulance, taken to A & E and admitted to hospital
ID09	NFA	UK	✓	PWID	✓			Hepatitis C	Partner called ambulance: hospitalised, discharged, readmitted to hospital & discharged. Later re-admitted when visited GP & told TB clinic trying to contact him
ID10	NFA	Ethiopia							GP made referral to neurologist & patient was hospitalised
	Hostel								
ID11	NFA, sometimes stays at a friend’s house	UK				✓		Stomach ulcers	GP prescribed a course of general antibiotics & later sent patient to hospital
ID12	B & B	Somalia							GP referral to hospital with letter via TB clinic
ID13	NFA	Ireland				✓		Arthritis	Taken to hospital by ambulance, & admitted via A & E
ID14	Hostel	UK	✓	PWID	✓	✓	✓		Doctor at DDU. Client says he went to four different hospitals but tests came back negative
ID15	Temporary bedsit	UK	✓	PWID	✓		✓	Hepatitis	Initially diagnosed in hospital for HIV related condition. Self discharged & later self referred through A & E and readmitted to hospital
ID16	Hostel	UK		PWID	✓				Hostel workers took him to A & E
ID17	B & B	Somalia							No GP, self referred through A & E

NFA no fixed abode usually sleeping on streets; PWID person who injects drugs; PWUD person who uses drugs; OST Opioid substitution therapy (methadone); B&B an individual room to sleep where breakfast is sometimes offered. MXU mobile x-ray unit; DDU Drug dependency unit; + did not have one main place of residence but would alternate.

### Drug and alcohol use

Nine (53%) participants reported drug use including polydrug use. Heroin, crack cocaine and valium were the main drugs used. Seven participants were receiving opioid substitution therapy (methadone maintenance) at the time of interview. PWUD also reported consuming alcohol, but to different degrees. Three other participants reported problematic alcohol use but that they did not use drugs. Everyday routines were dominated to some degree by drug and alcohol consumption and for people who injected drugs (PWID), ways of financing their habit and preventing drug withdrawal syndrome.

### Experiences of violence and social exclusion

Many participants disclosed past experiences of violence, torture, physical and sexual abuse. Others spoke of lives dominated by a cycle of crime and drugs, including youth offending (‘I’ve been a thief even as a kid’; ‘This is all I’ve ever known, it’s all I’ve ever done, in and out of hospital using drugs’; ‘I’ve been on the streets fifteen years on and off’). Three women had been victims of crime, including sexual violence in two cases, and physical assault resulting in a miscarriage in another. Two men spent time in prison during the course of their treatment. One young female, an undocumented migrant, spoke of the physical abuse and neglect at the hands of a family member, her guardian. Another participant reported that he was born to parents who used drugs and spent his life in and out of care homes. Others had migrated and fled civil unrest in their home countries. Patients’ experiences were therefore firmly embedded within narratives of violence and exclusion [27] and, in some cases, illustrated the cumulative effect of disadvantage which also shaped exposure to risk and behavioural choices [15,16].

### Personal accounts of health

Prior to their diagnosis of TB most interviewees described themselves as having good health with no history of illness. This mirrors the findings of survey research involving the general population [28]. On further questioning however, interviewees disclosed other health related issues e.g. HIV, epilepsy, hypertension, diabetes, a history of gastric ulcers (see Table 1). It is likely that some participants had mental health issues arising from drug use, or psychological trauma for those coming from war zones, or depression but patients did not volunteer this information.

Participants constructed illness in functional terms; that is, an inability to carry out every day activities such as shopping and cleaning or the need for a sick note, or hospitalisation (‘healthy, never hospitalised’). Only one participant volunteered that he didn’t look after himself while others reported a range of healthy behaviours including: diet (eating yoghurt or fruit) and exercise (working out or walking associated with being homeless or drug use). One Somali woman defined her health as poor in relation to another chronic illness, diabetes, for which she was receiving treatment. Many reported that a lack of income and stable housing affected their ability to eat healthily. Reliance on takeaways, because of limited access to cooking facilities, proved expensive and represented a large proportion of participants’ income. Some reported stealing food from shops or relying on ‘hand outs’ from the charity runs.

### Knowledge of TB and personal susceptibility

Most interviewees had heard of TB and, in some cases, knew others who either had the disease (often family member, friend or relative) or had died of TB. Participants located the risk of contracting TB either inside (opportunistic infection in the case of HIV) or outside the person (‘virulent strains coming from Asia’, ‘illegal immigrants’, ‘strong virus’). Other reasons included: personal responsibility, such as a failure to take medication for a previous (untreated) episode of TB; poor living conditions or environments (‘living on the streets’, ‘living in a dump, digs’, ‘living in a hostel where people used to spit in the corridor and TV rooms’) or lifestyle factors either associated with drug use (‘mixing in circles where people inject and smoke crack’) or unhealthy lifestyles (‘smoking dog ends’, ‘drinking’ ). Reasons given by those born overseas however were more likely to discuss the risk of exposure in relation to close contacts (i.e. family, friend or relative) or contact with a health care worker or setting. Other reasons were attributed to HIV, fatalism (given by God) or poor diet due to a lack of money. Not all attributions were correct however and, in general, knowledge of personal susceptibility in this group was low. TB was what happened to other people.

### Recognising symptoms

Historically, TB awareness raising and control efforts have emphasised the cardinal symptoms associated with pulmonary TB. A systematic review of international studies reported the following estimates of frequency of symptoms based on 25 studies: cough 85%, fever 65%, weight loss 62%, fatigue 55%, chest symptoms 50%, sweating 35%, blood in sputum 25% [29]. In this study participants reported a range of symptoms they had experienced typically associated with a diagnosis of tuberculosis. Common symptoms included tiredness, sweating, cough, loss of appetite, headaches, lethargy, shortness of breath, pain or ache, feeling cold and weight loss:

*I came to hospital my stomach felt full. Thought it was stomach*… *When I came to hospital I told them I felt tired*, *told them about stomach*, *saliva was salty tastes just a little saliva*, *sweating fever at night*, *temperature. This was in the summer in April*, *felt like this February to April* [ID17].

However participants commonly attributed their symptoms to other (undiagnosed) illnesses such as cancer, flu, or pre-existing conditions such as arthritis (‘pain in bones’) and pneumonia (difficulties breathing, chest pain). One participant stated that he was healthy before his diagnosis and mistook his symptoms of pain for physical exertion due to ‘working out’ to keep healthy. Moreover, although some patients did accurately report some of the symptoms of TB, their own illness narratives drew on metaphors to describe the severity of symptoms such as: ‘feeling drained’; ‘it’s like somebody stick a pipe in your bloodstream and sucking every bit of blood out of you’ [ID02]. The metaphor resonates with nineteenth century American folklore which associated TB with vampires who were believed to be responsible for the ‘wasting away’ of the consumptive patient [30]. Similarly the narrative of one homeless man discussing the context of his experience of night sweats and lethargy (sweat pouring off him despite sleeping in a draughty room during the winter months) provides rich contextual detail:

*In the evenings*, *you know waking up in this sweat would be pouring out of me and I think my first diagnosis*, *I think it was the January or the February I think it was January um it was cold*, *my window doesn*’*t close properly in my room*, *so my room is always cold. Do you know what I mean* (*pause*) *and yet I*’*m waking up in*, *I*’*m waking up all every hour or so and I*’*m pouring with sweat you know and feeling really lethargic and um you know*, *I*’*ve got no go in me whatsoever* [ID01].

Others experienced specific symptoms associated with non-pulmonary TB (NPTB) such as lumps, abscesses; one patient described a sense of numbness, loss of feeling, one sided weakness and fitting; another spoke of: ‘Stomach ache, back ache, rarely coughed’ (ID03). One patient delayed in seeking health care for five months despite the pain caused by an abscess in his neck. He believed the abscess was due to the cold weather and treated himself with paracetemol. He realised he had TB when the abscess burst.

Rather than have any specific or common symptoms patients reported a general sense of feeling unwell which they further explained in terms of tiredness, stomach pains and back ache. Some didn’t recall any symptoms while others reported symptoms not typical of tuberculosis, such as a difficulty swallowing or memory problems (which could have been due to alcohol consumption). One homeless man for example interpreted, and hence rationalised, his symptoms in the context of his everyday life and (mis) attributed his symptoms to drug withdrawal:

*I didn*’*t have a clue*....*sweating at night I put down to alcohol*, *the coughing down to smoking and um feeling unwell*, *down to withdrawal from um*, *the heroin* [ID05].

Rapid weight loss and loss of appetite are symptoms of TB but are also common experiences when using drugs. Stimulant drugs can suppress appetite and speed up the metabolism with the result that people may lose their appetite and experience weight loss. One participant recognised the weight loss despite a perception that he was eating a lot:

*I was doing heroin*, *and I was doing crack. I was doing all sorts all sorts of other drugs. And I was*, *eating*, *eating*, *eating*, *and*, *no way was I putting on weight*, *I was actually seven stone* [ID14].

Older migrant groups, particularly those from Somalia, had a greater awareness of TB but they did not always associate their symptoms with the disease. For example, a Somali refugee woman with no entitlement to welfare (apart from food vouchers^a^) attributed one of her symptoms, tiredness, to her diet but underpinning this was a lack of finance to buy good ‘halal’ food. The vouchers she received as part of welfare support were shop specific which limited her choice of food purchases. She also attributed her lethargy to her diabetes and ulcer.

These examples aptly illustrate the way the experience of illness and interpretation of symptoms are affected by the social context which can provide alternative, although in these examples, erroneous and misleading explanations for feeling unwell. Symptoms were either confused with drug use or drug withdrawal, or attributed to other illnesses or poor diet due to economic reasons.

### Accessing health care

For some, the first point of contact with health services was the General Practitioner (GP) who then either arranged immediate admission to hospital, attempted to treat with a course of general antibiotics or, where this had been unsuccessful, referred patients to the TB clinic. Only four patients did not have a GP at the start of treatment, attributed to the many specialist homeless health services in the catchment area of the clinic. However there were examples of missed or delayed diagnosis in primary care. This is not surprising, perhaps, given research has shown that primary care professionals underestimate the rates of TB within their own areas [31]. Diagnosis was also made during in-patient treatment for other conditions, notably HIV, although in one case the patient self-discharged before the results of the tests. Several people however accessed medical help only when they reached crisis point and were taken to hospital by ambulance after collapsing or through self-referral to urgent care, the Accident and Emergency Department (A & E). Smith et al. [32] concluded from their research that A & E was an important access point for TB patients and, therefore, also for screening initiatives and staff training.

In some cases it was only the intervention of friends, a partner, hostel worker or a member of the public (for example when patients collapsed in a public place) that enabled people to access care. One participant ‘blacked out’ on a bus and the police called an ambulance to take him to A & E. Another collapsed in a hostel and care workers took him to hospital suspecting TB after a recent training course aimed at raising awareness. One man accessed health care through a TB mobile x-ray screening initiative targeted at homeless hostels. One Somalian man missed three hospital appointments, prior to diagnosis, because he had left the family home due to marital breakdown which had left him homeless.

Even with severe symptoms, for example coughing up blood, one man delayed seeking medical advice attributing his symptoms to a rupture in his chest, his drug use or pneumonia, for which he was already receiving treatment:

*I ain*’*t got a clue. That was the last thing on my mind*, *TB*, *I*, *I just thought I was having pneumonia trouble. I thought I*’*d just ruptured something in me chest or something*… *I didn*’*t have a clue what it was* [ID09].

In this example the interviewee increased his drug use to deal with the pain of his symptoms and attempted to self-manage his condition:

*I was still getting like pains and all that in*, *my right side and all that*, *and I was thinking to myself*, *something ain*’*t right*, *something ain*’*t right. Or*, *you know*, *I was just taking drugs*: *to kill the pain*. …*You know what I mean*? *Go on having a fix and*, *the more fixes you were having*, *the less pain you have. Cos of*, *opiate*’*s a morphine based substance so*, *it killed a lot of the pain* [ID09].

It was only when his partner urged him to go to hospital that he was admitted through A & E where he was later diagnosed with tuberculosis. Prior to this he had been discharged from hospital three times with a diagnosis of pneumonia, although it was unclear whether he discharged himself or was discharged by medical staff. A male refugee also reported frequent visits to a primary care practitioner, his GP, three to four times a month for sickness notes. He self-referred to A & E with pain and difficulty eating and following several tests, was diagnosed with NPTB and admitted to hospital.

### Barriers to seeking care

Drug dependency and the issue of ‘scoring drugs’ dominated every day routines for PWID. One patient co-infected with HIV described his life in terms of a cycle of begging to procure drugs, injecting and begging again to prevent drug withdrawal syndrome. The prospect of hospitalisation was a major barrier to seeking help for fear of having little control over his drug withdrawal:

*Cos the reason I never came into* ‘*ospital*, *was because I was I was on heroin and like I I thought*, *well if I go into* ‘*ospital they*’*ll keep me in*, *I*’*m gonna withdraw I I didn*’*t know*, *that they give methadone and all that*, *you know*, *not everybody knows that*, *you know* [ID06].

Another homeless drug user however reported that admission to hospital was a welcome break from the strain of living on the street offering the prospect of a ‘roof,’ a bed and a television. One participant interviewed while in prison stated that inmates would be deterred from seeking health care as they would be admitted to the hospital wing of the prison, declared unfit for work and, deprived of a valuable source of income to buy cigarettes^b^ (‘The monetary aspect is enough to keep it a secret’, ID01), although this was not his personal experience. Additionally he felt his chances of moving to another prison were reduced while receiving medical care and attending hospital appointments. This was particularly undesirable in a ‘harsh’ prison environment where much of the day was spent confined to a cell:

*You*’*re locked up all the time. It*’*s the worst prison*; *people want to get shipped out. If you have hospital appointments you won*’*t get shipped out*, *you won*’*t tell them you*’*ve got TB if you suspect* [ID01].

### Managing risk to access care

There were examples of rather creative and strategic approaches to managing the risk of drug withdrawal and seeking medical attention. One woman recounted how she obtained a sufficient quantity of money to buy enough drugs to sustain a long (anticipated) period of waiting in A & E. In this example the woman calculated that a bag of heroin would provide 10–12 hours free from drug withdrawal symptoms after which she would be admitted to hospital where she would receive methadone therapy. An inability to manage her drug withdrawal with no means of alleviating the symptoms, she reasoned, would result in her leaving the hospital without receiving medical care; in short, a wasted journey. In this example the journey to the hospital was for painful leg ulcers (she was unaware she had TB):

*Anyway this w*’ *when was it Tuesday night and I just. I had the money to score my heroin to be able to keep me*, *in*, *the A & E Department until I was to be admitted and to get my methadone. You see that was my main concern. I didn*’*t want to be in a situation where*, *I*’*d maybe be in there mayb*’, *I dunno*, *maybe ten or twelve hours and start withdrawing*, *not have any heroin and not have any money to score it. Which would then mean I wasted twelve hours* [ID15].

The woman rationalised that God must have willed her admittance to hospital based on a number of chance events: obtaining the money, obtaining the drugs (‘the dealer was there’) and the availability of transport (‘caught the bus’) to A&E.

## Conclusions

A consensus statement on the treatment of TB in urban risk groups in big cities recommended the need to: “identify barriers and promote access to healthcare services for all those at risk of TB” [33]. The outcome of this study has highlighted some of the individual and institutional barriers to accessing care and treatment. Much of TB prevention focuses on the need to raise awareness through the recognition of symptoms. Tuberculosis awareness raising activities in particular are premised on educational models which assume that once informed, human beings will behave rationally, and act accordingly. However our research suggested that the relationship between symptom recognition and seeking help was neither linear nor automatic as patients tended to normalise their symptoms in the context of their everyday lives. Since symptoms were attributed to causes for which participants would not ordinarily seek medical attention, such as drug and alcohol withdrawal, tiredness attributed to poor diet for economic reasons, and other long term conditions, indicates the ease with which serious illness can be missed by those living with complex health and social issues. For this reason, perhaps, the TB stigma reported in other studies as a barrier to health care [34] was not cited as a factor (although it was discussed in terms of a fear of disclosure of illness to others in the Somalian community by one Somali refugee). Our research supports the intensification of outreach initiatives to identify groups at risk of TB and the development of structured care pathways to support people into prompt diagnosis and treatment.

### Reflections on research

Participants interviewed in this study represent some of the most marginalised in our communities. This presented challenges when researching the views of PWUD because of drug withdrawal, intoxication and mood swings. One drawback of the study was the use of clinical interpreters, rather than bilingual interviewers with training in research, which may have influenced how some of the interviews were conducted. The resulting transcripts were best described as summaries, rather than translated texts, and were therefore less ‘data rich’. There is scant research reporting on the experience of translators working with vulnerable groups or how translation ‘shapes meaning making’ [35]. Resources, training and support for bilingual interviewers will need to be factored into research grants which could prove costly in areas where clinical caseloads are linguistically diverse.

Ethical concerns that vulnerable patients may feel coerced into taking part in research studies based in clinics where patients are receiving their care are important to consider [24]. Patients may also participate in research in anticipation of personal gain. For example after one interview, an older Somali refugee asked if she would be entitled to a house. This suggested she had constructed the research interview as another assessment of her immigration status and, hence, eligibility for housing even though the researcher, and the participant information sheet, made it clear that there was no personal benefit to taking part in the study. Finally, some interviewees discussed matters that were distressing for the researcher, for example violence (as perpetrators or survivors). This illustrates the need for protocols on disclosure, guidelines on safety and support for researchers and staff working with vulnerable groups, in addition to referral pathways to agencies with skills to support research participants.

## Endnotes

^a^Since this study was conducted the voucher system has been abolished.

^b^Prisoners are eligible for sick pay but at a reduced rate compared to earnings.

## Competing interest

The authors declare that they have no competing interests.

## Authors’ contributions

GC was the lead researcher and interviewed participants, analysed data, developed analytical frameworks and drafted the manuscript. LJ independently corroborated data analysis and drafted sections of the manuscript. AZ was the project director and was involved in the design of the study and contributed to the writing of the manuscript. All authors read and approved the final version.

## Pre-publication history

The pre-publication history for this paper can be accessed here:

http://www.biomedcentral.com/1471-2458/14/618/prepub
